# Assessment of Subclinical Cardiac Alterations and Atrial Electromechanical Delay by Tissue Doppler Echocardiography in Patients with Nonfunctioning Adrenal Incidentaloma

**DOI:** 10.5935/abc.20180188

**Published:** 2018-11

**Authors:** Gulizar Sokmen, Murat Sahin, Dilek Tuzun, Abdullah Sokmen, Hanife Bolat, Ayten Oguz, Adem Doganer, Huseyin Nacar, Kamile Gul

**Affiliations:** 1 Kahramanmaras Sutcu Imam University, Faculty of Medicine, Department of Cardiology, Kahramanmaraş - Turquia; 2 Kahramanmaras Sutcu Imam University, Faculty of Medicine, Department of Internal Medicine, Kahramanmaraş - Turquia; 3 Kahramanmaras Sutcu Imam University, Faculty of Medicine, Department of Endocrinology, Kahramanmaraş - Turquia; 4 Kahramanmaras Sutcu Imam University, Faculty of Medicine, Department of Biostatistics and Medical Informatics, Kahramanmaraş - Turquia

**Keywords:** Incidental Findings, Diastole/function, Adrenocortical Adenoma, Diagnostic ,Imaging, Metabolic Syndrome, Cardiac Conduction System Disease

## Abstract

**Background:**

Majority of the incidentally discovered adrenal masses, called adrenal incidentaloma (AI), are nonfunctioning adrenal adenomas. The appropriate management of AI is still a matter debate, so it is necessary to investigate their associated morbidity. However, data regarding morphological and functional cardiac alterations are limited in this group.

**Objective:**

In this study, we aimed to assess cardiac structural and functional characteristics and atrial conduction properties in patients with nonfunctioning AI.

**Methods:**

Thirty patients with nonfunctioning AI and 46 properly matched control subjects were included in the study. After hormonal and biochemical analysis, all participants underwent transthoracic echocardiography to obtain systolic and diastolic parameters of both ventricles, in addition to atrial conduction times by tissue Doppler echocardiography. Data were analyzed with Statistical Package for the Social Sciences (SPSS, Chicago, IL, United States) statistics, version 17.0 for Windows. P < 0.05 was considered statistically significant.

**Results:**

Left ventricular (LV) mass index and LV myocardial performance index were significantly increased in AI group. Among atrial conduction times, both intra- and interatrial electromechanical delays were significantly prolonged in patients with nonfunctioning AI. Other laboratory and echocardiographic findings were similar between groups.

**Conclusion:**

Our study revealed that intra- and inter-atrial conduction times were prolonged, and LV mass index was increased in patients with nonfunctioning AI. These findings may be markers of subclinical cardiac involvement and tendency to cardiovascular complications. Close follow-up is necessary for individuals with nonfunctioning AI for their increased cardiovascular risk.

## Introduction

Adrenal incidentaloma (AI) is defined as an adrenal mass, generally discovered in radiological interventions for indications other than adrenal disease. The classic definition excludes patients with clinically overt adrenal hormone secretion, and those with concurrent malignancy, known as metastasis to the adrenals. The prevalence of adrenal masses in general population has been reported to be as high as 6% at autopsy, and 2.5-4.2% on evaluation of abdomen and thorax by computerized tomography (CT).^1^ Majority of the incidentally discovered adrenal masses are nonfunctioning adrenal adenomas.^2^ The appropriate management of these patients is still a matter of debate, and it is necessary to investigate their associated morbidity. The presence of AI has been proposed as a new cause of metabolic syndrome and reported to increase cardiovascular disease risk. The burden of disturbances showed diversity from impaired glucose tolerance to increased epicardial fat thickness and intima-media thickness of common carotid arteries.^2^^-^^6^ However, data regarding morphological and functional cardiac alterations are still limited in this particular group.

Atrial fibrillation (AF) is one of the most common arrhythmias observed in clinical practice. Several electrocardiographic and echocardiographic markers reflecting electrophysiological and electromechanical abnormalities of atria prone to develop AF have been studied with the aim of early identification of patients susceptible to develop AF. Atrial electromechanical delay (EMD) has been defined as the temporal delay between the onset of electrical activity and the mechanical activation of atrial myocardium. Tissue Doppler echocardiography (TDE) is a simple, noninvasive and reliable method to measure atrial EMD.^7^ Several studies report that atrial EMD measured by TDE is a valuable parameter to predict new onset AF or recurrence of AF.^8^^-^^11^

There are few studies evaluating cardiac functions in patients having nonfunctioning AI, but to our knowledge data concerning atrial electromechanical properties are lacking.^5^^,^^12^ The aim of this study was to assess intra- and inter-atrial conduction times along with cardiac structural and functional characteristics in patients with nonfunctioning AI.

## Methods

### Study population

All subjects who were referred to the Department of Endocrinology and Metabolism of Kahramanmaras Sutcu Imam University, in Kahramanmaras, Turkey, with incidentally discovered adrenal tumors between March 2014 and November 2015 were recorded (n = 82). The study was approved by local Research Ethics Committee of the institution in compliance with the Declaration of Helsinki. All of the participants gave written consent.

At the first visit, all subjects underwent a CT or *magnetic resonance imaging* (MRI) scan to confirm the diagnosis. Adrenal adenoma was diagnosed if the following criteria were met:

- tumor size less than 4.0 cm;
- regular shape with well-defined margins;
- homogenous and attenuation value of 10 or less Hounsfield units on unenhanced CT scan, and 30 or less Hounsfield units on enhanced CT scan.^13^

After confirming the presence of adrenal adenoma, detailed physical examination and basal hormonal and dynamic tests were performed. Among participants, 30 patients having nonfunctional adrenal adenomas were included in the study. Totally 52 patients with uncompleted tests or hormone-secreting tumors - high levels of dehydroepiandrosterone sulphate (DHEAS), hyperaldosteronism, Cushing syndrome (CS), pheochromocytoma - and patients having large adrenal tumors (>4 cm) with irregular borders and invasion to adjacent structures raising the suspicion of malignancy were excluded. Additionally, patients with known malignancies, coronary artery disease, valvular heart diseases, cardiomyopathies, thyroid dysfunction, chronic renal failure, liver failure, previous surrenal or hypophysial intervention or patients taking steroids were not included in the study. In an attempt to determine the hormonal activity of adrenal tumors, blood samples were collected at 8.30 am to analyze sodium (Na), potassium (K), adrenocorticotropic hormone (ACTH), DHEAS and plasma cortisol levels. For exclusion of CS, 1 mg dexamethasone suppression test (DST), the most common screening test for CS, was performed to the patients. The suppression in overnight DST was adequate when morning cortisol level fell below 1.8 µ/dl. Blood samples for plasma renin and aldosterone measurements were collected after at least 4-6 hours of nocturnal resting, following two hours of standing or wandering, and after 15 minutes of resting, respectively. Plasma aldosterone concentration (PAC)/plasma renin activity (PRA) ratio was used as the screening test to exclude primary aldosteronism. PAC/PRA ≤ 20 values were accepted as normal.^14^ Moreover, patients were given a diet free of food containing phenolic acid for five days. Then, 24-hour urine specimens were collected for metanephrine and normetanephrine analyses. Pheochromocytoma was defined as elevated levels of urinary normetanephrine (normal range: 88-444 µg/day) and/or urinary metanephrine (normal range: 52-341 µg/day).^13^ Age and sex matched 46 subjects without clinical suspicion of hypercortisolism, with normal DHEAS level (female 35-430 µg/dl, male 80-560 µg/dl), suppressed 1 mg DST (≤ 1.8 µg/dl) and without adrenal mass on abdominal ultrasonography were taken as the control group. Blood pressure measurements of all subjects were taken from right arm in the sitting position after 5 minutes of resting. Height (meter) and weights (kg) of all participants were recorded, and body mass index (BMI, kg/m^2^) was calculated.

### Conventional echocardiographic examination

All participants were performed transthoracic echocardiography (Vivid 7 Pro, GE, Horten, Norway, 2-4 MHz phased array transducer), including two-dimensional, M-mode, pulsed, and color flow Doppler examinations by the same experienced cardiologist blinded to the clinical status of the subjects. Recordings were made on left lateral decubitus position by using standard parasternal, apical, and subcostal views. Left atrial dimension, left ventricular (LV) end-diastolic and end-systolic diameters, diastolic thickness of ventricular septum and posterior wall were measured from M-mode in parasternal long axis view according to the criteria of the American Society of Echocardiography guidelines. The early (E-wave) and late diastolic (A-wave) velocities of mitral inflow were measured from apical four chamber view with pulsed Doppler echocardiography by placing the sample volume at the tips of mitral leaflets, and E/A ratio was calculated. Ejection fraction was estimated by Simpson’s rule. LV mass was calculated by Devereux formula and indexed to the body surface area.^15^^-^^17^

Right ventricular (RV) morphological and functional parameters including right atrial dimension, RV diameter, and tricuspid annular plane systolic excursion (TAPSE) were measured according to the American Society of Echocardiography guidelines.^15^ Systolic pulmonary artery pressure (sPAP) was obtained from the maximum velocity of the regurgitant tricuspid jet, and pulmonary acceleration time (PAT) was measured as the time between the onset and peak of pulmonary velocity obtained by pulsed Doppler recording.^18^

### Tissue doppler echocardiography and atrial electromechanical delay

TDE was performed with transducer frequencies of 3.5-4.0 MHz using a 5 mm pulsed Doppler sample volume. Spectral Doppler signal filters were set to obtain a Nyquist limit of 15 to 20 cm/s with minimal optimal gain settings. The sweep speed was set at 50 to 100 mm/s. A single lead electrocardiogram (ECG) was recorded simultaneously during measurements. In the apical four chamber view, the sample volume was subsequently placed at the level of LV lateral mitral annulus, septal mitral annulus and RV tricuspid annulus. The sampling window was positioned as parallel as possible to the myocardial segment of interest to obtain the optimal angle of imaging. Time intervals from the onset of P wave on the surface ECG to the beginning of the A wave (PA) representing atrial EMD were obtained from lateral mitral annulus, septal mitral annulus, and tricuspid annulus and named PA lateral, PA septum and PA tricuspid, respectively. The difference between PA lateral and PA tricuspid was defined as inter-atrial EMD (PA lateral-PA tricuspid), the difference between PA lateral and PA septum was defined as intra-atrial EMD (PA lateral-PA septum). Peak systolic (Sm), early diastolic (Em), late diastolic (Am) velocities, and isovolumic contraction time (ICTm; time interval between the end of Am and the beginning of Sm), isovolumic relaxation time (IRTm; time interval between the end of Sm and the beginning of Em), and ejection time (ETm; time interval between the beginning and the end of Sm) were obtained from mitral and tricuspid annulus. Em/Am ratio for both ventricle and E/Em for LV were calculated. The myocardial performance index (MPI), a noninvasive Doppler measurement of global ventricular function incorporating both systolic and diastolic function, was calculated by the formula of (ICTm+IRTm)/ETm for both ventricles.

### Reproducibility

Intraobserver variability was assessed in 20 subjects randomly chosen from the participants, and the echocardiographic measurements were repeated under the same basal conditions. Simple random sampling method was used in the selection of 20 subjects. 1,96*(S_w_/√2n(m-1)) = confidence in the estimate formula was used to estimate the sample size for reproducibility. Reproducibility was evaluated by coefficient of variation. Intraobserver coefficients of variation were found to be nonsignificant (< 5%).

### Statistical Analysis

Data were analyzed with Statistical Package for the Social Sciences (SPSS, Chicago, IL, United States) statistics, version 17.0 for Windows. Shapiro-Wilk test was used to test the normality of distribution for continuous variables. Continuous variables were expressed as means ± standard deviation. Non-normal distributed variables were expressed as Median and quartiles (1.Quartile-3.Quartile). Categorical data were presented as numbers and percentages. Difference between groups was detected using χ^2^ test for categorical variables. Mean values of continuous variables were compared between groups using Independent samples t-test or Mann-Whitney U-test, according to whether normally distributed or not. Correlation between continuous variables was evaluated by Pearson correlation tests. A linear regression analysis and generalized linear models were used to identify predictors of atrial EMD. P < 0.05 was considered statistically significant.

## Results

Clinical and laboratory data of the study groups are given in Table 1. Age, gender, BMI, systolic and diastolic pressures, heart rate, and ratio of diabetic and hypertensive subjects were similar between groups (p > 0.05). ACTH and DHEAS levels were significantly lower in nonfunctioning AI group (p = 0.009 and p < 0.001, respectively). Cortisol levels were similar, but suppression with 1 mg DST was pronounced significantly in the control group (p < 0.001). Other laboratory data including fasting plasma glucose, low-density lipoprotein (LDL) cholesterol, high-density lipoprotein (HDL) cholesterol, triglyceride and insulin levels did not differ between groups.

**Table 1:** Baseline characteristics of the study population

Characteristics	Non-functioning AI (n = 30)	Control (n = 46)	p value
Age^a^ (years)	51.77 ± 8.23	50.80 ± 6.62	0.46
Female sex^c^, n (%)	25 (83.3)	41 (89.1)	0.84
BMI^a^ (kg/m^2^)	34.30 ± 4.63	32.43 ± 3,93	0.07
Diabetes mellitus^c^, n ( %)	3 (10)	6 (13)	0.76
Hypertension^c^, n (%)	5 (16.7)	8 (17.4)	0.94
DM and Hypertension^c^, n (%)	5 (16.7)	7 (15.2)	0.89
Systolic blood pressure^a^ (mmHg)	131.33 ± 16.49	125.85 ± 12.36	0.07
Diastolic blood pressure^a^ (mmHg)	81.57 ± 10.36	78.46 ± 10.62	0.21
Heart rate^a^ (bpm)	82.93 ± 13.00	77.72 ± 9.19	0.09
Cortisol^a^ (µg/dl)	12.88 ± 2.94	11.71 ± 3.80	0.15
Post DST cortisol^a^ (µg/dl)	1.11 ± 0.38	0.70 ± 0.26	< 0.001*
ACTH^b^ (pg/ml) Median (Q1-Q3)	14,70(12,50–20,30)	22,40(13,70–35,70)	0.009*
DHEAS^b^ (µg/dl) Median (Q1-Q3)	55,15(27,90–86,30)	113(73,80–157,00)	< 0.001*
Fasting plasma glucose^b^ (mg/dl) Median (Q1-Q3)	98(87,00–111,00)	97(84,00–113,00)	0.61
LDL cholesterol^b^ (mg/dl) Median (Q1-Q3)	101,45(91,00–123,70)	109(91,90–135,00)	0.56
HDL cholesterol^a^ (mg/dl)	45.50 ± 9.10	45.57 ± 9.49	0.97
Triglycerides^b^ (mg/dl) *Median (Q1-Q3)*	116,50(84,00–153,00)	142(105,00–235,00)	0.1

a Independent samples t test;

b Mann-Whitney U test;

c χ^2^ test;

* difference is statistically significant; AI: adrenal incidentaloma; BMI: body mass index; DM: diabetes mellitus; DST: dexamethasone supression test; ACTH: adrenocorticotrophic hormone; DHEAS: dedhydroepiandrostenedione sulphate; LDL: low-density lipoprotein; HDL: high-density lipoprotein.

Conventional echocardiographic parameters were shown in Table 2. There were no significant differences between groups considering LV end-diastolic and end-systolic diameters, LV ejection fraction, diameter of left and right atrium, RV diameter, TAPSE and systolic PAP. Diastolic thickness of interventricular septum (IVS), posterior wall (PW) and LV mass index was significantly higher (p = 0.03, p = 0.03, and p = 0.004 respectively), and PAT was significantly lower (p = 0.004) in nonfunctioning AI group. Although mitral E/A ratio was lower in nonfunctioning AI compared to the control group, the difference was not statistically significant (p = 0.07).

**Table 2:** Comparison of conventional echocardiographic parameters between groups

Variable	Non-functioning AI (n = 30)	Control (n = 46)	p value
LV end-diastolic diameter^a^ (mm)	48.83 ± 3.70	46.93 ± 3.64	0.07
LV end-systolic diameter^b^ (mm) Median (Q1-Q3)	27(26,00–28,00)	27(25,00–30,00)	0.96
LV ejection fraction^a^ (%)	71.93 ± 7.54	72.26 ± 5.84	0.68
IVS diastolic thickness^b^ (mm) Median (Q1-Q3)	10(9,00–11,00)	9(8,00–11,00)	0.03*
PW diastolic thickness^b^ (mm) Median(Q1-Q3)	11(9,00–12,00)	10(9,00–11,00)	0.03*
LV mass index^a^ (gr/m^2^)	112.01 ± 26.93	95.33 ± 21.69	0.004*
Left atrial diameter^a^ (mm)	36.27 ± 2.79	35.59 ± 2.84	0.31
Mitral E/A ratio^a^	0.87 ± 0.25	1.01 ± 0.30	0.07
RV basal diameter^a^ (mm)	32.14 ± 3.54	32.74 ± 3.91	0.51
RA diameter^a^ (mm)	32.20 ± 4.71	32.61 ± 3.98	0.69
TAPSE^b^ (mm) Median (Q1-Q3)	24(20,00–26,00)	22,50(21,00–27,00)	0.42
sPAP^a^ (mmHg)	25.67 ± 3.45	26.11 ± 3.92	0.65
PAT^a^ (ms)	96.38 ± 22.08	113.48 ± 26.36	0.004*

a Independent samples t test;

b Mann-Whitney U test; Median (Q1-Q3): Median (1.Quartile-3.Quartile);

* difference is statistically significant; AI: adrenal incidentaloma; LV: left ventricular; IVS: interventricular septum; PW: posterior wall; RV: right ventricular; RA: right atrial; TAPSE: tricuspid annular plane systolic excursion; sPAP: systolic pulmonary artery pressure; PAT: pulmonary acceleration time.

Comparison of tissue Doppler parameters and atrial conduction times were demonstrated in Table 3. LV lateral, LV septal, global LV Em/Am and RV Em/Am were decreased significantly in nonfunctioning AI group (p = 0.02, p = 0.03, p = 0.01, and p = 0.004, respectively). LV septal MPI and LV MPI were significantly higher in nonfunctioning AI group (p = 0.004 and p = 0.03, respectively), whereas LV lateral and RV MPI did not differ significantly between groups. There was no significant difference between groups with regard to Sm and E/Em. PA lateral, PA septum and PA tricuspid were not different between groups. Inter-atrial EMD and intra-atrial EMD were significantly higher in nonfunctioning AI group compared to the controls (p = 0.008 and p = 0.016, respectively).

**Table 3:** Comparison of tissue Doppler parameters and atrial conduction times between groups

Variable	Non-functioning AI (n = 30)	Control (n = 46)	p value
**LV lateral annulus**			
Sm^b^ (cm/s) Median (Q1-Q3)	9(8,00–11,00)	10(8,00–11,00)	0.39
Em/Am^b^ Median (Q1-Q3)	0,72(0,62–1,00)	0,93(0,79–1,20)	0.02*
E/Em^b^ Median (Q1-Q3)	6,77(5,29–8,33)	6,82(5,50–7,46)	0.52
MPI^b^ Median (Q1-Q3)	0,44(0,39–0,53)	0,46(0,42–,52)	0.81
**LV septal annulus**			
Sm^a^ (cm/s)	8.80 ± 2.11	8.37 ± 1.43	0.43
Em/Am^b^ Median (Q1-Q3)	0,64(0,55–0,83)	0,73(0,63–1,00)	0.03*
E/Em^a^	10.16 ± 3.36	10.18 ± 2.22	0.77
MPI^a^	0.52 ± 0.07	0.47 ± 0.11	0.004*
**RV tricuspid annulus**			
Sm^a^ (cm/s)	15.57 ± 3.57	14.35 ± 2.77	0.11
Em/Am^b^ Median (Q1-Q3)	0,58(0,46–0,67)	0,67(0,60–0,81)	0.004*
MPI^a^	0.46 ± 0.06	0.43 ± 0.10	0.11
LV Sm^b^ (cm/s) Median (Q1-Q3)	9,00(7,50–10,00)	9,00(8,00–10,50)	0.96
LV Em/Am^a^	0.76 ± 0.24	0.88 ± 0.22	0.01*
LV E/Em^b^ Median (Q1-Q3)	7,85(6,25–10,00)	8,15(6,79–9,29)	0.90
LV MPI^a^	0.50 ± 0.05	0.47 ± 0.12	0.03*
**Atrial conduction times**			
Lateral PA^a^ (ms)	45.97 ± 10.95	42.35 ± 8.16	0.09
Septum PA^a^ (ms)	30.87 ± 9.86	31.11 ± 7.21	0.78
Tricuspid PA^b^ (ms) Median (Q1-Q3)	21,00(18,00–26,00)	22,00(18,00–26,00)	0.34
Intra-atrial EMD^a^ (ms)	15.10 ± 7.97	11.24 ± 4.08	0.016*
Inter-atrial EMD^a^ (ms)	23.53 ± 7.99	18.85 ± 5.79	0.008*

a Independent samples t test;

b Mann-Whitney U test; Median (Q1-Q3): Median (1.Quartile-3Quartile);

* difference is statistically significant; AI: adrenal incidentaloma; LV: left ventricular; MPI: myocardial performance index; RV: right ventricular; PA: time interval from the onset of P wave on electrocardiogram (ECG) to the beginning of the A wave; EMD: electromechanical delay.

Bivariate correlation analysis revealed that inter-atrial EMD was negatively correlated with ACTH level (r = -0.29, p = 0.027), mitral E/A ratio (r = -0.33, p = 0.004), and RV Em/Am ratio (r = -0.29, p = 0.011), and positively correlated with LV mass index (r = 0.38, p = 0.001), left atrial diameter (r = 0.23, p = 0.04), age (r = 0.32, p = 0.004) and systolic blood pressure (r = 0.23, p = 0.04). Intra-atrial EMD was positively correlated with post DST cortisol level (r = 0.23, p = 0.04), LV mass index (r = 0.33, p = 0.004), age (r = 0.34, p = 0.003) and systolic blood pressure (r = 0.32, p = 0.004), and negatively correlated with mitral E/A ratio (r = -0.36, p = 0.002). Multivariate relationships of inter- and intra-atrial EMD with clinical parameters revealed that changes in post DST cortisol levels affected intra-atrial EMD significantly (Wald χ^2^= 3.810, p = 0.049) (Table 4). We also found that increase of post DST cortisol level by 1 µg/dl lengthened intra-atrial EMD by 4.752 msec.

**Table 4:** Assessment of subtle cortisol secretion related effects on intra-atrial electromechanical delay (EMD)

			Hypothesis Test	
Parameter	B	Standard Error	Wald Chi-square	p
Intercept	13.121	4,0795	10.345	0.001
Post DST cortisol	4.752	2.4347	3.810	0.049*
Cortisol	-0.265	0.2642	1.004	0.316
ACTH	-0.090	0.0725	1.551	0.213
DHEAS	0.008	0.0167	0.258	0.611

Generalized Linear Models; α: 0,05;

* effect is statistically significant; DST: dexamathasone supression test; ACTH: adrenocorticotrophic hormone; DHEAS: dedhydroepiandrostenedione sulphate.

## Discussion

This is the first tissue Doppler echocardiographic study evaluating abnormalities of atrial conduction together with cardiac structure and function in nonfunctional adrenal incidentalomas. We obtained two important findings:

- LV mass increased significantly;
- Intra- and inter-atrial conduction times were delayed significantly in these patients.

It is well known that overt cortisol excess, as in Cushing syndrome, may lead to systemic complications responsible for increased cardiovascular risk (hypertension, obesity, impaired glucose metabolism, dyslipidemia) and cardiovascular complications such as coronary heart disease and congestive heart failure.^6^^,^^19^ It has also been shown previously that Cushing syndrome causes cardiac structural changes associated with LV dysfunction.^20^^,^^21^ However, it is still a matter of debate whether nonfunctional AI increases the risk of cardiovascular disease and whether this type of adrenal tumor has some degree of autonomous adrenal function. In this study, we obtained some indirect evidence of subtle cortisol autonomy and cardiovascular risk in patients with nonfunctioning AI. There are few studies analyzing cardiac morphology and function in nonfunctional AI. Ermetici et al.^12^ reported the presence of LV hypertrophy and LV diastolic dysfunction in patients with nonfunctional AI.^12^ Iacobellis et al.^5^ showed increased epicardial fat thickness and LV mass by transthoracic echocardiography in these subjects.^5^ Similarly, we found that LV mass index was increased significantly in patients with nonfunctional AI compared to the control group. The impact of LV hypertrophy on cardiac mortality and morbidity has been understood increasingly.^16^ It has been suggested that cortisol production by AI may have a broad spectrum, ranging from normal to various degrees of excess daily production rate, and this may not be detectable by standard endocrine work-up.^12^ In our study, basal cortisol levels of the groups were similar, but post DST cortisol levels were significantly elevated (not exceeding the cut-off, 1.8 µg/dl), and DHEAS levels were significantly reduced (not below the cut-off, 40 µg/dl) in nonfunctioning AI group. Additionally, post DST cortisol level was correlated with LV mass index. According to these findings, we speculated that subtle cortisol autonomy of adrenal adenoma might play a role in cardiac hypertrophy.

Myocardial performance index is a parameter calculated from tissue Doppler echocardiographic measurements, and predicts both systolic and diastolic ventricular function. In our study, LV MPI was found to be increased in patients with AI indicating impaired global LV function. This impairment may be attributed largely to the impairment of LV diastolic function, because the predictors of LV systolic function such as LV EF and LV Sm were similar in both groups.

Considering structural and functional parameters of RV, decreased RV Em/Am ratio might indicate the tendency to impairment of RV diastolic function. PAT was also shortened, indicating increased pulmonary vascular resistance in patients with AI.

Atrial fibrillation is the most common arrhythmia encountered in clinical practice, and associated with significant mortality and morbidity due to hemodynamic impairment and thromboembolic events. Impaired atrial conduction is an important step in the pathophysiology of AF. Atrial conduction times can be evaluated by both invasive (electrophysiological study) and noninvasive (P wave dispersion on ECG and EMD on echocardiography) methods.^22^ It has been shown that impaired atrial conduction is an independent and strong predictor for development and recurrence of AF, and TDE is a useful and reliable technique to evaluate atrial electromechanical properties.^7^^-^^9^ Numerous studies demonstrated that atrial conduction time was prolonged in various diseases including obesity, thyroid diseases, chronic obstructive lung diseases, non-alcoholic fatty liver disease, acromegaly and diabetes mellitus (DM).^23^^-^^28^ Cushing disease is associated with many cardiovascular risk factors, including glucose intolerance, hypertension, LV hypertrophy, central obesity and metabolic syndrome, and may lead to cardiovascular events such as coronary heart disease, heart failure and arrhythmias.^21^ So, we hypothesized that AI might be associated with cardiac structural and functional changes, and increased risk of AF. Earlier studies showed increased epicardial fat, increased LV mass and LV diastolic dysfunction in AI similar to our results.^5^^,^^12^ However, they did not study atrial conduction properties in this patient group.

Therefore, this study showed for the first time that both intra- and inter-atrial EMD were impaired in patients with nonfunctioning AI. Moreover, atrial EMD was correlated significantly with post DST cortisol level, ACTH level, LV mass index, LV diastolic dysfunction, age and systolic blood pressure. Post DST cortisol level was an important predictor of intra-atrial EMD, such that 1 µg/dl increase in post DST cortisol level caused the prolongation of intra-atrial EMD by 4.752 msec. We may explain these findings by a few mechanisms. First, subtle cortisol excretion can affect cardiac structure and function as mentioned previously, which in turn is supposed to have detrimental effects on atrial conduction. Secondly, AI and AF share common metabolic risk factors such as increased blood pressure, insulin resistance, endothelial dysfunction and obesity. Lastly, low-level but long-standing subtle cortisol excretion may have direct toxic effect on myocardium by glucocorticoid receptors leading to myocardial fibrosis.^4^^-^^6^^,^^13^^,^^29^ Detection of prolonged atrial EMD in these patients may be an earlier sign of atrial dysfunction preceding AF.

### Study limitations

The major limitation of the study was the relatively small number of the subjects in adenoma group, besides the inability to define the length of the disease owing to the lack of overt clinical features. Finally, our study lacks long-term follow-up data, since it is a cross-sectional study. The patients could not be followed for future arrhythmic episodes to see whether the ones with prolonged atrial EMD develop AF.

## Conclusion

Our study revealed that intra- and inter-atrial conduction times were prolonged and LV mass index was increased in patients with nonfunctioning AI. These findings may be markers of subclinical cardiac involvement and tendency to cardiovascular complications. Thus, individuals diagnosed to have nonfunctioning AI should be followed up closely for their increased cardiovascular risk.
